# The Cost of Provisions in Institutions.—VII

**Published:** 1904-06-04

**Authors:** 


### HOSPITAL ADMINISTRATION.

### CONSTRUCTION AND ECONOMICS.

### THE COST OF PROVISIONS IN INSTITUTIONS.—VII.

BY THE AUTHOR OF "HOSPITAL EXPENDITURE—THE COMMISSARIAT."

(*Continued from page* 140.)

ST. THOMAS'S HOSPITAL (NUMBER OF BEDS, 544).

THE cost of provisions per bed at St. Thomas's Hospital during the last five years was as follows:—

| Year | Average of occupied beds | Average cost per bed |
|---|---|---|
| | | £ |
| 1902 ... ... ... | 465 | 24 |
| 1901 ... ... ... | 443 | 24 |
| 1900 ... ... ... | 419 | 24 |
| 1899 ... ... ... | 400 | 24½ |
| 1898 ... ... ... | 407 | 23¾ |

This amount represents, it will be remembered, the cost of boarding all the inmates of the hospital divided by the average number of patients in residence.

The board of the patient, always the most easily reckoned item in the hospital household because the exact number of days they are in residence is ascertainable, is 8⅓d. a day, or £12 13s. a year. Deducting the cost of the patients' board from the total cost of provisions, and dividing the remainder among the available beds, we have the following figures for the last three years:—

| Year | Number of occupied beds out of 544 available | Total cost of provisions | Cost of patient, at £12 13s. a year | Average cost of provisions, exclusive of patients | |
|---|---|---|---|---|---|
| | | | | Per occupied bed | Per available bed |
| | | £ | £ | £ | £ |
| 1902 | 465 | 11,098 | 5,882 | 11⅕ | 9½ |
| 1901 | 443 | 10,624 | 5,604 | 11⅓ | 9¼ |
| 1900 | 419 | 10,033 | 5,300 | 11⅓ | 8¾ |

It has been before explained that an institution household is necessarily maintained in accordance with its maximum capacity, and that the number of the staff is not appreciably affected by fluctuations in the number of patients received in any given year. Hence, in comparing the average cost of victualling the hospital one year with another, a more accurate result is often obtained by taking for a basis the number of available beds.

The numbers other than patients boarded at St. Thomas's Hospital are as follows:—

| — | Numbers | Average of occupied beds to each | Average of available beds to each |
|---|---|---|---|
| Medical officers ... | 15 | 27 | 36 |
| Nursing staff, including Nightingale probationers | 165 | 2·4 | 3·2 |
| Female servants ... | 61 | 6·6 | 9 |
| Male servants ... | 3 | — | — |
| Total ... ... | 244 | 1·6 | 2·2 |

The board of the Nightingale probationers, 45 in number, is included in the general account, but the cost is entered among receipts from the Nightingale Fund per contra. These numbers are for 1903, and although they vary slightly from time to time, they have been practically the same for the last year or two.

The percentage other than patients is 33 of the total number of inmates.

The cost of the nurses' board is 8s. 10d. a week, or about £23 a year. The cost of the nurse's maintenance is officially reckoned at 1s. 6d. a day, but this amount includes 2s. weekly for laundry.

The cost of the officers' board is 3s. a day, including wine, or £55 a year.

The totals, reckoned on this basis, and allowing for servants' board at 5s. a week, work out at practically the same as the actual amount spent on provisions in 1902.

The cost of the inmates as enumerated works out as follows:—

| | £ |
|---|---|
| Average of 465 in-patients at 8⅓d. a day ... | 5,893 |
| „ 165 nurses at £23 a year ... ... | 3,795 |
| „ 15 medical officers at £55 a year... | 825 |
| „ (64 servants, say, at £13 a year)... | 832 |
| | £11,345 |
| Amount expended on provisions ... ... | £11,360 |

There are, perhaps, not many hospital managers who succeed in keeping so well within the limits of their estimates as these figures indicate.

We append an analysis of the published accounts showing the average consumption for each article under the head of provisions, per bed and per inmate:—

| | |
|---|---|
| Daily average of patients ... ... ... | 465 |
| Daily average of "persons other than patients" | 244 |
| Total daily average boarded in hospital ... | 709 |

| — | Total cost | Average per occupied bed | Average per head |
|---|---|---|---|
| | £ | £ s. | £ s. |
| Meat ... ... ... | 3,696 | 7 18 | 5 4 |
| Fish and poultry ... | 1,054 | 2 5 | 1 9 |
| Butter and cheese ... | 1,245 | 2 13 | 1 15 |
| Eggs ... ... ... | 335 | 0 14 | 0 9 |
| Milk ... ... ... | 2,067 | 4 8 | 2 18 |
| Bread, flour, etc. ... | 698 | 1 10 | 0 19 |
| Grocery ... ... | 1,513 | 3 5 | 2 2 |
| Vegetables ... ... | 487 | 1 0 | 0 13 |
| Malt liquor ... ... | 262 | 0 11 | 0 7 |
| Ice and mineral waters ... | 97 | 0 4 patients only | |
| Wine and spirits ... | 145 | 0 6 patients only | |

The patients' diet includes tea, sugar, and butter. Bread is supplied *ad lib.*, except in fever diet. There are three varieties of full diet, four of middle diet, and two of fever diet, differing in kind and quantity. The most substantial form of diet allows 6 oz. of cooked meat (4 oz. for female patients), 4 oz. of potatoes or green vegetables, 4 oz. of batter or suet pudding. Pure fever diet consists of 3 pints of milk, or milk with gruel, barley water, mutton broth, or beef tea, as ordered.

Extras are served on the signed order, renewed at each regular visit, of the physician or surgeon, and returns of the extras ordered are circulated periodically to those responsible for ordering them.

The average number of those on milk or fever diet is 51, 10 per cent. of the total average of in-patients.

The amount of meat required for making beef tea is about 600 lb. a week, and about 870 lb. of meat are required weekly in joints. Roughly speaking, about 75,000 lb. of meat are used in the hospital in a year, and the importance of this branch of the commissariat is at once apparent.

The quantity of milk required for the patients is 800 gallons a week. This works out at just under 14 pints a week each, or about a quart a day.

Separate contracts for milk are made half-yearly, there being a difference of 2d. a gallon between winter and summer price. The milk is frequently tested, and a high standard of quality is ensured.

The total consumption of milk in the hospital amounts to 168 gallons a day, or over 60,000 gallons a year. A difference of only one penny a gallon would cause a rise in the milk bill of £250 a year. It is only by incessant watchfulness that a low contract for milk can be made to prove satisfactory; but without such watchfulness, no matter what price is paid for the milk, its quality will not be uniformly satisfactory.

It cannot be too often reiterated that it is the maintenance of good quality in all the food supplies which should be the leading consideration in the hospital commissariat, and the experience of St. Thomas's Hospital goes to prove that a low average of expenditure follows hard on the good marketing and skilled supervision essential to good results.

